# Candesartan Does Not Activate PPARγ and Its Target Genes in Early Gestation Trophoblasts

**DOI:** 10.3390/ijms232012326

**Published:** 2022-10-14

**Authors:** Lena Neuper, Daniel Kummer, Désirée Forstner, Jacqueline Guettler, Nassim Ghaffari-Tabrizi-Wizsy, Cornelius Fischer, Herbert Juch, Olivia Nonn, Martin Gauster

**Affiliations:** 1 Division of Cell Biology, Histology and Embryology, Gottfried Schatz Research Center, Medical University of Graz, 8010 Graz, Austria; 2 Division of Immunology, Otto Loewi Research Center for Vascular Biology, Immunology and Inflammation, Medical University of Graz, 8010 Graz, Austria; 3 Institute for Medical Systems Biology (BIMSB), 10115 Berlin, Germany; 4 Diagnostic and Research Institute for Human Genetics, Diagnostic and Research Center for Molecular BioMedicine, Medical University of Graz, 8010 Graz, Austria; 5 Experimental and Clinical Research Center, a Cooperation between the Max-Delbrück-Center for Molecular Medicine in the Helmholtz Association and the Charité—Universitätsmedizin Berlin, 13125 Berlin, Germany; 6 Charité—Universitätsmedizin Berlin, Corporate Member of Freie Universität Berlin and Humboldt—Universität zu Berlin, 10117 Berlin, Germany; 7 Max-Delbrück-Center for Molecular Medicine in the Helmholtz Association (MDC), 13125 Berlin, Germany

**Keywords:** candesartan, PPARγ, placenta, trophoblast, first-trimester pregnancy, rosiglitazone, teratogenic, angiotensin II

## Abstract

Angiotensin II receptor 1 blockers are commonly used to treat hypertension in women of childbearing age. While the fetotoxic effects of these drugs in the second and third trimesters of pregnancy are well documented, their possible impacts on placenta development in early gestation are unknown. Candesartan, a member of this group, also acts as a peroxisome proliferator-activated receptor gamma (PPARγ) agonist, a key regulator shown to be important for placental development. We have previously shown that trophoblasts do not express the candesartan target–receptor angiotensin II type 1 receptor AGTR1. This study investigated the possible role of candesartan on trophoblastic PPARγ and its hallmark target genes in early gestation. Candesartan did not affect the PPARγ protein expression or nuclear translocation of PPARγ. To mimic extravillous trophoblasts (EVTs) and cytotrophoblast/syncytiotrophoblast (CTB/SCT) responses to candesartan, we used trophoblast cell models BeWo (for CTB/SCT) and SGHPL-4 (EVT) cells as well as placental explants. In vitro, the RT-qPCR analysis showed no effect of candesartan treatment on PPARγ target genes in BeWo or SGHPL-4 cells. Treatment with positive control rosiglitazone, another PPARγ agonist, led to decreased expressions of *LEP* and *PPARG1* in BeWo cells and an increased expression of PPARG1 in SGHPL-4 cells. Our previous data showed early gestation–placental AGTR1 expression in fetal myofibroblasts only. In a CAM assay, AGTR1 was stimulated with angiotensin II and showed increased on-plant vessel outgrowth. These results suggest candesartan does not negatively affect PPARγ or its target genes in human trophoblasts. More likely, candesartan from maternal serum may first act on fetal-placental AGTR1 and influence angiogenesis in the placenta, warranting further research.

## 1. Introduction

It is increasingly acknowledged that chronic hypertension has become a global epidemic in recent decades [1]. When lifestyle changes are no longer sufficient to normalize blood pressure, a prescription of appropriate medications is required. Angiotensin receptor blockers (ARBs) are commonly used medications that can be used in monotherapy. ARBs have some advantages, such as fewer side effects, the possibility of combination therapy, and potential cardioprotective effects. Furthermore, they might be more effective in the younger population and are often prescribed when patients do not tolerate therapy with angiotensin-converting enzyme (ACE) blockers, another popular blood pressure medication [2]. Candesartan is an angiotensin II receptor type I (AGTR1) blocker commonly used to treat hypertension [1,2]. In 2019, 3.2% of women in Austria under the age of 45 suffered from hypertension [3]; thus, when women with chronic hypertension plan to become pregnant, they should receive medical advice from their resident gynecologists for possible changes in their antihypertensive therapies, especially if their treatments include ACEs or ARBs since both medications have proven to be teratogenic [4,5,6,7]. It has been associated with fetotoxicity in pregnant patients, including oliguria, oligohydramnios, pathological development of fetal lungs, perinatal renal failure, and fetal death [4,5,6,7]. Of note, there is no evidence of embryotoxic effects of candesartan intake in the first trimester, according to small studies addressing this issue [8,9]. These findings are in line with studies on first trimester exposure to other AGTR1 blockers and angiotensin-converting enzyme inhibitors (ACE inhibitors), which were also shown to be teratogenic in the second and third trimester, but not in the first trimester [6,10]. Prescriptions of ACEs and ARBs are often switched to beta-blockers, methyldopa, or labetalol, which is a combined alpha-blocker and beta-blocker [11]. Education about blood pressure also plays an important role in preconception counseling since many chronic hypertensive women are not aware of their conditions. This often leads to the masking effect of pre-existing hypertension in the first trimester, because the blood pressure physiologically lowers during that period [11]. During pregnancy, some changes in blood pressure are normal. Pre-existing hypertension, defined as hypertension that is present before conception or hypertension, manifests within the first 20 weeks of gestation [12]. Up to 10% of all pregnancies are affected by hypertensive disorders, which are a cause for increased mortality and morbidity of the mother and the fetus. In about 3% of pregnancies, preeclampsia occurs [13], and chronic hypertension accounts for 5–10% of those cases but increases the odds ratio by 3.8–17.1-fold [14,15]. Preeclampsia is defined as a new onset of hypertension with proteinuria or a new onset of hypertension without proteinuria but with end-organ dysfunction. When preeclamptic patients additionally experience seizures without any previous neurological disorders, it is called eclampsia [16]. Eclampsia occurs in 0.2–0.5% of pregnancies and leads to a 2% rise in maternal mortality and 12% in the fetus [17].

Notwithstanding the risks and efforts to change blood pressure medication through pre-pregnancy counseling, it is still likely that women with hypertension and ACEs or ARB treatments become pregnant. In particular, as it is estimated that 40% of pregnancies worldwide are unintended [18], women who have conceived unknowingly or unintentionally may have no chance to change to safe treatments in early pregnancy. With an increasing prevalence of chronic hypertension around the world, blood pressure medication use rises [11]. This also elevates the chances that women who are knowingly or still unknowingly pregnant will take medications, such as candesartan. While there are some limited clinical studies on AGTR1 therapy, including candesartan in pregnancy, focusing on the fetotoxic effects, there are very few studies on how candesartan affects the development and function of the placenta early in gestation before fetal organs are fully developed. We shed light on potential adverse mechanisms of candesartan in human trophoblasts possibly affecting embryonic development, fetal-maternal crosstalk, and fetal nutrition early in gestation.

**Figure 1:**
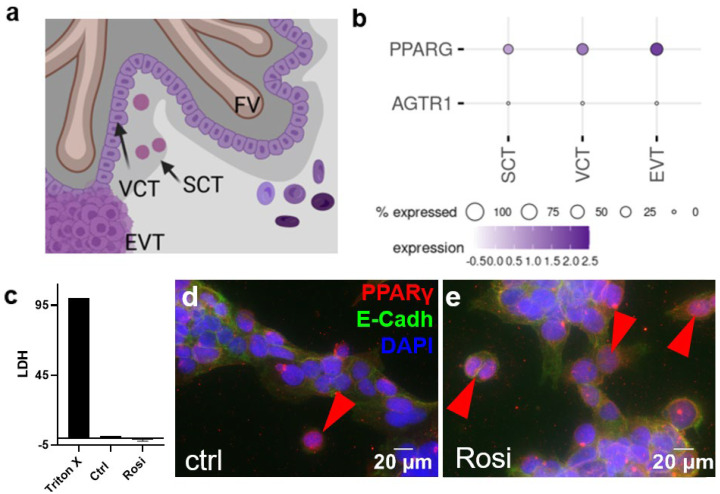
(**a**) Schematic drawing of trophoblast subpopulations assessed by single-cell RNA-sequencing in panel b. (**b**) Expression pattern of possible binding sites for AGTR1-blocker candesartan at the maternal–fetal interface in human first-trimester placenta (single-cell RNA sequencing data from Vento-Tormo et al. published 2018 in *Nature*) [33]. (**c**) The LDH assay was performed with the supernatants of BeWo cells treated with 5 µM of rosiglitazone for 24 h and the respective controls. BeWo cells were stained for E-cadherin (green) and PPARγ (red). Vehicle control- (DMSO 0.5% *v/v*) (**d**) and rosiglitazone- (5 µM) (**e**) treated BeWo cells showed few PPARγ-stained nuclei after 24 h incubation. A double immunofluorescence staining with E-cadherin for trophoblast cell–cell contacts and PPARγ showed PPARγ-positive nuclei in the controls (arrowheads).

The placenta is the organ that represents the connection between the mother and the embryo/fetus during pregnancy. It is the placental villi that are in direct contact with the maternal blood and, thus, with all nutrients, metabolites, and, in the case of maternal drug intake, pharmaceuticals. The outermost layer of these villi in contact with maternal blood is the syncytiotrophoblast (SCT), which is formed from the proliferation, differentiation, and fusion with underlying villous cytotrophoblasts (VCT). Another type of trophoblast is the extravillous trophoblast (EVT), which migrates into the maternal decidua and replaces the endothelium of maternal spiral arteries during the development of the placenta (Figure 1a). The correct formation of the placenta and the differentiation of the various subpopulations of trophoblasts are essential for a normal and healthy pregnancy [19].

Amongst others, the peroxisome proliferation-activated receptor (PPAR)γ plays a fundamental role in the development and proper functioning of the placenta. Mostly the isoform PPARγ1 is found in villous and extravillous trophoblasts and a knockout resulted in the termination of pregnancy in mice. Studies have also shown that agonists, such as rosiglitazone, inhibit trophoblast invasions in primary human trophoblast cells [20]. Rosiglitazone also altered the expression of the human chorionic gonadotropin beta subunit (β-hCG) in villous and extravillous trophoblasts. Furthermore, placentas from preeclamptic pregnancies have been shown to have lower PPARγ expressions, as PPARγ is affected by hypoxia [21]. This also has an effect on SCT differentiation and trophoblast apoptosis, in which PPARγ is also involved [21]. The PPARs belong to the steroid receptor superfamily comprising the three isoforms PPARα, PPARβ/δ, and PPARγ. The PPARs play important roles in various differentiation and metabolic processes, mainly in glucose and lipid balance and adipocyte differentiation [22]. After being activated, the receptors relocate to the nucleus, form a heterodimer with the retinoid X receptor (RXR), and initiate gene regulations of their target genes [22,23]. Obesity, type 2 diabetes, and arteriosclerosis are some diseases that are linked to the dysregulation of PPARγ. The receptor has been detected in adipose tissue, the spleen, and small intestines, with adipocytes showing the highest expression [22]. PPARγ expression was also found in placental tissue [23].

Another group of PPARγ agonists is the sartan family [24,25]; previous research shows the effects of different sartans on PPARγ on different cell types and cell lines and the effects of candesartan on a mouse brain (in which traumatic brain injuries could be healed better), rat epididymal adipose tissue (which was more sensitive to insulin after candesartan treatment), and liver cells (which showed a possible new therapy for metabolic syndrome) [25,26,27,28]. The effects in humans have also been described in clinical studies about metabolism, pharmacokinetics, drug–food interactions, drug–drug interactions, influences of age, gender, ethnicity, and various diseases. However, there have been no studies conducted on how candesartan impacts placental development [24]. Nevertheless, studies exist showing the fetotoxicity of candesartan. They found that maternal treatment with candesartan led to the pathological development of fetal lungs, skulls, and kidneys, as well as oligohydramnios [29,30,31]. 

In this study, we aimed to determine if candesartan affects the early developing human placenta and which effects these are. Therefore, we examined the effect of candesartan in the villous trophoblast compartment that interacts with the maternal circulation, i.e., the SCT, and the trophoblasts invading maternal uterine tissue, the EVT.

## 2. Results

In order to determine how candesartan interacts with the early human placenta, we first analyzed, which of the three major trophoblast subpopulations (Figure 1a) express the candesartan target receptors *AGTR1* and *PPARG*. In a previous study, we have shown that SCT and VCT displayed a lack of expression for the classical candesartan target receptor *AGTR1* [32]. Single-cell data from a previous study showed that they expressed a further candesartan-target *PPARG*, with EVTs having the highest expressions and SCT the lowest expression (Figure 1b) [33]. Since BeWo cells represent a model for the villous trophoblast compartment, this cell line was used for this study [34,35]. In initial experiments, BeWo cells were treated with 5 µM rosiglitazone for 24 h in order to study the activation of PPARγ. To evaluate toxic effects, an LDH assay to test for cytotoxicity was performed. The assay showed that rosiglitazone, at the concentration used, was not cytotoxic for the cells, compared to the Triton X treatment (positive controls, Figure 1c). To test if PPARγ was activated in response to rosiglitazone treatment, cell nuclei were assessed for PPARγ staining, where the transition of the receptor from the cytoplasm to the nucleus was a sign of its activation. Staining for PPARγ suggested a basal activation of the receptor under control conditions (Figure 1d), which was, however, not significantly affected by rosiglitazone treatment (Figure 1e). 

**Figure 2:**
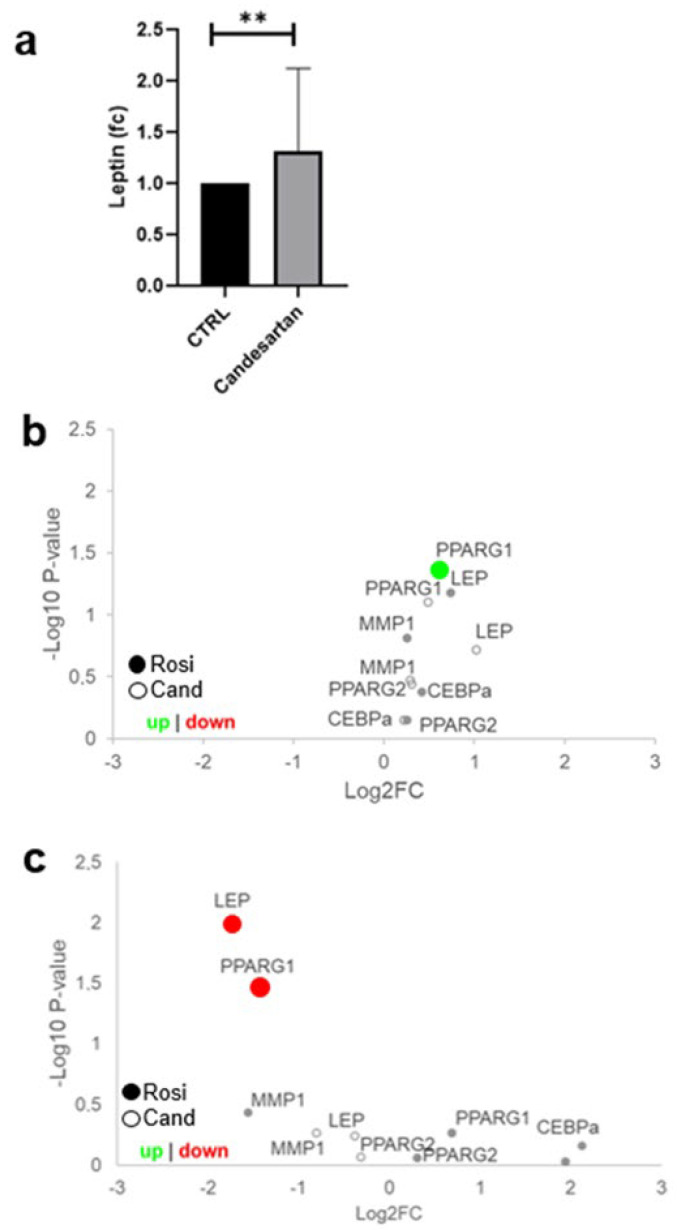
**Leptin secretion is upregulated by candesartan in placental explants.** (**a**) First-trimester placental villous explants were cultured for 24 h in 0.1 µM candesartan (Cand) and control conditions (*n* = 8, isovolumetric control with DMSO). Leptin concentrations were upregulated 1.5-fold (one-sample *t*-test, *p* = 0.0025). (**b**) SGHPL-4 cells were treated with 5 µM rosiglitazone or 1 µM candesartan. In qPCR, *PPARG1* was upregulated in rosiglitazone-treated cells (*n* = 3, Kruskal–Wallis *p* = 0.0210). (**c**) BeWo cells were treated with 5 µM rosiglitazone (Rosi) or 1 µM candesartan (Cand) for 48 h. qPCR analysis showed that *LEP* (*n* = 3, Unpaired *T*-test, ** *p* = 0.0099) and *PPARG1* (*n* = 3, Unpaired *T*-test, *p* = 0.0345) were downregulated in rosiglitazone treated cells.

To show the effects of candesartan on the early human placenta, first-trimester placental villous explants were cultured with 0.1 µM candesartan for 24 h. This concentration was chosen according to previously described human pharmacokinetics in vivo after treatment with candesartan [36]. The candesartan treatment resulted in a 1.5-fold higher leptin secretion into the supernatants, compared to DMSO-treated explant controls (Figure 2a, one-sample *t*-test, *p* = 0.0025). While BeWo cells represent a model of VCT/SCT, the SGPHL-4 cell line represents a model of EVT [34,35]. To cover all possible interaction points of candesartan and rosiglitazone, both cell lines were used in this study. Since we could not activate PPARγ target genes with 0.1 µM candesartan, we increased the concentration to 1 µM for the experiments with BeWo and SGHPL-4 cells [37]. When BeWo cells were treated under the same conditions as SGHPL-4 cells, rosiglitazone led to a downregulation of *LEP* (Figure 2c, Unpaired *T*-test, *p* = 0.0099) and *PPARG1* (Figure 2c, Unpaired *T*-test, *p* = 0.0345). On the other hand, when treated with 1 µM candesartan or 5 µM rosiglitazone for 48 h, SGHPL-4 cells showed a significant upregulation of *PPARG1* in rosiglitazone treated cells (Figure 2b, Kruskal–Wallis *p* = 0.0210). In contrast, candesartan did not affect the target genes in both cell lines.

**Figure 3:**
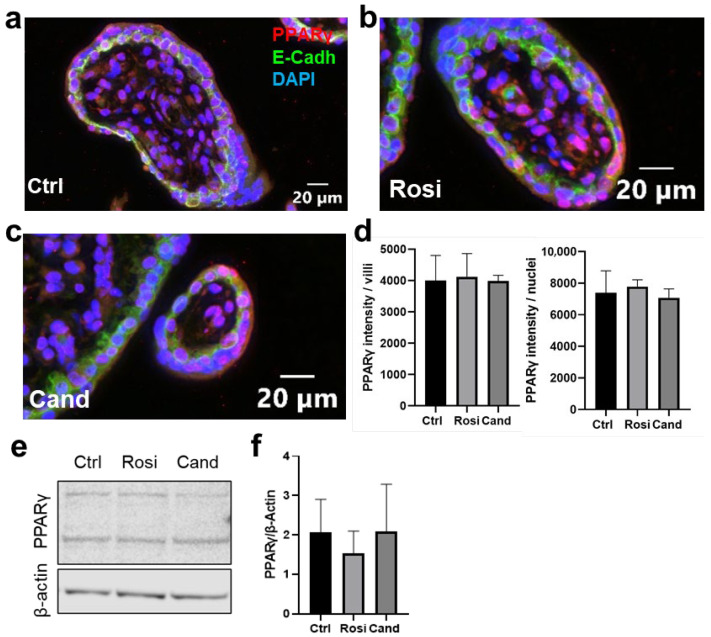
**Candesartan has no effect on PPARγ nuclear translocation in trophoblasts of first-trimester placenta explants.** First-trimester placenta explants were treated with (**a**) solvent control (DMSO, ctrl), (**b**) 100 µM rosiglitazone (Rosi), or (**c**) 10 µM candesartan (Cand) for 24 h. Stainings are shown on the control (Ctrl), rosiglitazone, and candesartan-treated explants. Nuclei counterstained with DAPI (blue), tissue stained with E-Cadherin (green), and PPARγ (red). (**d**) Rosiglitazone and candesartan treatment did not affect the intensity of PPARγ intensity on trophoblast cytoplasmic and nuclear areas compared to the control (*n* = 4, one-way ANOVA, *p* = 0.6465). The treatments also did not affect the PPARγ intensity over the villous area (*n* = 4, one-way ANOVA, *p* = 0.9596). (**e**) Western blot analysis of first-trimester explants treated with 100 µM rosiglitazone or 10 µM candesartan for PPARγ showed two bands. Only the lower ban was used for the analysis at 54–57 kDa. (**f**) Rosiglitazone and candesartan treatments did not affect the PPARγ protein expression (*n* = 4, one-way ANOVA, *p* = 0.6271).

The next step was to determine whether candesartan affected the protein level of PPAR**γ** in first-trimester placental villous explants. Since we could not show the activation of PPAR**γ** in previous experiments, we again increased candesartan and rosiglitazone concentrations to 10 and 100 µM, respectively, according to other described studies [38,39,40,41]. The explants were treated with DMSO as a solvent control, 100 µM rosiglitazone as a positive control, or 10 µM candesartan. To test the nuclear translocation of PPAR**γ**, tissue sections of the explants were then stained for E-cadherin and PPAR**γ** (Figure 3a–c). The software-based analysis of staining intensity showed that neither rosiglitazone nor candesartan had an effect on the overall PPARγ expression (intensity of PPARγ on the total villous surface; Figure 3d, *n* = 4, one-way ANOVA, *p* = 0.9596) or nuclear localization of PPARγ (intensity of the PPARγ staining per nuclei; Figure 3d, *n* = 4, one-way ANOVA, *p* = 0.6465) in the villous trophoblast compartment. In line with histological observations, the western blot analysis showed that neither rosiglitazone nor candesartan had an effect on PPARγ levels (Figure 3e, f, *n* = 4, one-way ANOVA, *p* = 0.6271).

**Figure 4:**
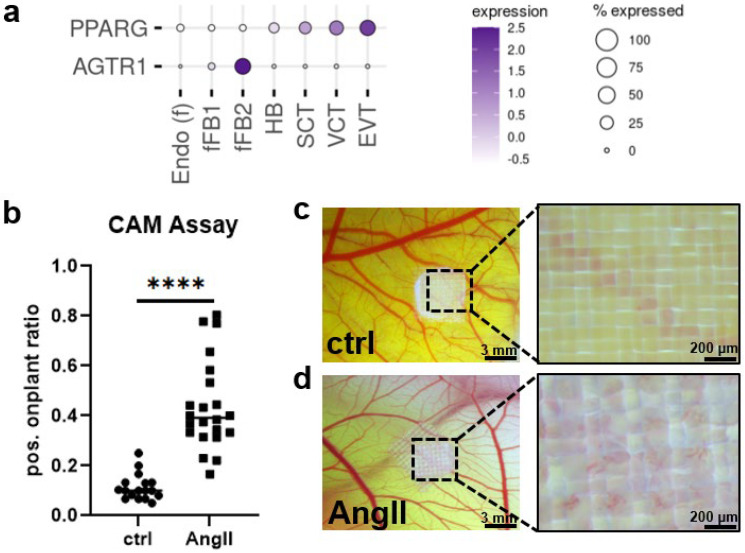
**Candesartan target AGTR1 is expressed in fetal myofibroblasts.** (**a**) Expression pattern of possible binding sites for AGTR1-blocker candesartan at the maternal–fetal interface in the human first trimester placenta (single-cell RNA sequencing data from Vento-Tormo et al. published 2018 in *Nature* [33]). (**b**) Chicken chorioallantoic membrane (CAM) assay showed significantly increased on-plant vessel outgrowths (*n* = 4, unpaired *t*-test, **** *p* < 0.0001; scale bar in left panels represents 3 mm, in right panels 200 µm) when treated with 0.1 µM angiotensin II (AngII) for 24 h (**d**) as compared to DMSO controls (**c**).

In summary, candesartan does not seem to have an adverse impact on PPARγ and its target genes in placental trophoblast subpopulations. Therefore, we extended our investigation to placental cell types, which were not in direct contact with maternal blood. Candesartan from maternal serum may pass the placenta and cross into the fetal-placental circulation [42]. We have previously shown that the candesartan target *AGTR1* is only expressed in fetal–placental myofibroblasts and not in trophoblasts (Figure 4a) [32,33]. Hence, candesartan may not interact with trophoblasts but could potentially affect blood vessel growth via the effects on early gestational placental myofibroblasts expressing *AGTR1*. We performed a chicken chorioallantoic membrane (CAM) assay to examine whether 0.1 µM angiotensin II (AngII) for 24 h had an effect on blood vessel growth in this model. The results showed that treatment with AngII increased on-plant vessel outgrowths compared to the respective DMSO control (Figure 4b, unpaired *t*-test, *p* < 0.0001).

## 3. Discussion

In summary, maternal candesartan intake can have severe consequences on fetal development [4,5,7]. As we have previously shown, the villous trophoblast compartment does not express the classical candesartan target AGTR1 but only PPARγ as another possible interaction site of candesartan [32]. PPARγ is essential for placental development in mice, as PPARγ null mice failed to form structured and functioning placentae in previous studies [43]. The mouse embryos died on day ten of pregnancy and the only PPARγ expression found in embryonic tissue of wild type mice at that point of pregnancy was in the placenta, which points to PPARγ having a key role in placental development [32]. Furthermore, a study showed that placental PPARγ expression seems to be vital for placental vascularization since PPARγ null placentas showed disarrayed vascular structures in mice. They also showed diminished differentiation of trophoblasts since no syncytium was formed. This indicates that the supply of nutrients to the embryo might be hampered [43]. Activation of PPARγ can also lead to reduced trophoblastic invasion in vitro [20,44]. Furthermore, PPARγ is involved in intracellular and transcellular lipid transfer, uptake, and storage [45,46,47]. 

Since the physiological candesartan concentration (0.1 µM) did not activate PPARγ, we increased the concentrations to supraphysiological levels based on previous literature in which cells were treated with candesartan. It has been described that the concentrations of some drugs might be different in the blood of pregnant women than in the cord blood or fetal blood [48,49,50]. This effect might be even bigger when it comes to drugs that have a high affinity for binding to plasma proteins [49]. Since sartans are known to pass the placental barrier [42] and over 90% of candesartan is bound to plasma protein in humans [24], it is possible that the candesartan concentration in the cord or fetal blood might be higher than in maternal blood. Nevertheless, we could not show the activation of PPARγ via treatment with candesartan, even though we used supraphysiological candesartan concentration, indicating that adverse effects of candesartan via PPARγ on human placental trophoblasts are unlikely. This would explain why clinical studies have not found convincing evidence for adverse effects of AGTR1 blockers on pregnancy outcomes after first-trimester exposure, although a potential pharmacologic target of known influence on early placental development, PPARγ, is expressed in trophoblast.

Of note, using the PPARγ agonist rosiglitazone as a positive control, we could activate the receptor to a certain degree in the trophoblast. Rosiglitazone led to an increased expression of *PPARG1* in BeWo cells and decreased expressions of *LEP* and *PPARG1* in SGHPL-4 cells. This is in line with the observation that treatment with PPARγ agonists, such as rosiglitazone, seem to speed up the process of trophoblast differentiation to the point of a too-small quantity of stem cells in mice [47]. Furthermore, rosiglitazone has an effect on human SCT differentiation and trophoblast apoptosis, in which PPARγ is also involved [51,52]. In human primary trophoblasts, troglitazone, another PPARγ agonist, led to the formation of a syncytium, which is an indicator of differentiation, whereas 15ΔPGJ2, another PPARγ agonist, led to highly reduced syncytialization [53]. On the other hand, troglitazone seems to prevent term trophoblast apoptosis due to acute hypoxia [52]. Furthermore, placentas from preeclamptic pregnancies have been shown to have lower PPARγ expressions, as PPARγ is affected by hypoxia [54,55]. The differentiation and invasion of trophoblasts appear to be influenced by PPARγ agonists. Previous data show that in human trophoblasts, activation of PPARγ with rosiglitazone and 15D-PGJ_2_ leads to reduced invasion in vitro [44]. However, our rosiglitazone results, in line with published data on similar drugs, indicate that further studies investigating the potential adverse effects of such commonly used oral anti-diabetic drugs on early placental development and functions are necessary.

Profiling of the target–receptor expression using single-cell RNA-sequencing [33] suggests that early gestational myofibroblasts expressing AGTR1 could respond to candesartan and cause adverse effects, including impaired placental angiogenesis. In the CAM model of early embryonic vascularization, the AGTR1-agonist angiotensin II induced changes in blood vessel outgrowth, indicating a possible influence of AGTR1 antagonists on this process as well. Of note, the reported adverse fetal effects of maternal candesartan treatment, such as renal failure, pulmonary hypoplasia, and skull hypoplasia, are remarkably similar to those reported for maternal treatment with ACE inhibitors [56]. These observations suggest that the pharmacological suppression of the fetal renin–angiotensin system (RAS) by either antagonizing the fetal AGTR1 or inhibiting the fetal ACE activity severely impairs fetal vascular perfusion and renal functions, presumably leading to mentioned malformations.

In summary, PPARγ can affect vascularization in the placenta of mice [43], with further studies underlining that candesartan can affect vascularization. In an ischemic retinopathy mouse model, candesartan treatment led to increased physiological vascularization with decreased pathological tufts. Furthermore, the retina was better re-vascularized by candesartan and it stimulated tip-cells while also blocking nitrative and oxidative stress [57]. There is also a study in which candesartan improved arterial stiffness and endothelial dysfunction in patients suffering from coronary artery disease, although patients with more severe manifestations responded better to treatment with candesartan than patients with milder manifestations [58]. Candesartan has also been described as neuroprotective after strokes in rat models with smaller infarct volumes than in control groups or groups treated with angiotensin-converting enzyme inhibitors [59]. Furthermore, rats show better vascular self-regulation and significantly smaller cerebral infarctions after pre-treatment with candesartan [60]. Lastly, candesartan has also been attributed to a role in neuronal regeneration in rats [61]. Doubtlessly, further studies are needed to investigate the potential effects of candesartan and other sartans on placental vascularization.

Even though we found no interaction of candesartan with trophoblastic PPARγ, which might be a positive sign for the safety of this drug during early placenta development, there is a great number of fetal morbidities and mortalities that can originate in maternal candesartan use. The majority of newborns had fetal RAS-blockade syndrome when mothers were administered ARBs during their pregnancies. Other complications associated with maternal ARB use are anuria, renal failure, oligohydramnios, respiratory stress syndrome, intrauterine dearth or miscarriage, limb defects, hypocalvaria, intrauterine growth restriction, arterial hypertension, pulmonary hypoplasia, and cerebral complications [4,5,6,7]. Taking all of this into account, it is apparent that general patient counseling on hypertension and the effects of certain medications on fetuses is important. Greater awareness of the general public, and especially of women of childbearing age, is needed to prevent these ARB-induced fetal morbidities. In addition, ACE inhibitors and AGTR1 antagonists are not the drugs of choice in women of childbearing age, due to well-known fetotoxicity. Therefore, inadvertent exposures and the resulting consultations of clinical teratologists, as bases for high-quality observational cohort studies [62], e.g., regularly performed by Teratology Information Services in Europe (ENTIS) and the US (OTIS), are not expected to be very common. It will probably take several years to collect a sufficient number of exposures and follow-up data for an observational cohort study sufficiently powered to statistically rule out at least a doubling of the basic risks for adverse pregnancy outcomes after first-trimester exposure to AGTR1 antagonists.

While PPARγ is essential for the proper development of the placenta and, thus, for the entire course of pregnancy, we could not find any association between candesartan and PPARγ activation in placental trophoblasts in this study. By profiling the target–receptor expression using single-cell RNA-sequencing [33], it seems likely that early gestational myofibroblasts expressing *AGTR1* could respond to candesartan and cause adverse effects, including impaired placental angiogenesis. 

## 4. Methods and Materials

### 4.1. Cell Culture

BeWo cells obtained from ECACC were used for cell culture experiments. The cells were cultured with DEMEM/F-12 (1:1) (1×) Dulbecco´s Modified Eagle (Gibco, cat. no. 15140-122, LOT: 2145455) and 5% L-Glutamine 200 mM (100×) (Gibco, cat. no. 25030-024, LOT: 1978718); 2.5 × 10^6^ cells were seeded with 15 mL medium in a T75 flask and incubated at 37 °C with 5% CO_2_. 

SGHPL-4 cells obtained from ECACC were cultured with DMEM (1×) Dulbecco´s Modified Eagle Medium (Gibco, cat. no. 31885-023, LOT: 1906052) containing 10% fetal bovine serum (HyClone^TM^, cat. no. SV30160.03, LOT: RB35939), 5% penicillin–streptomycin (Gibco, cat. no. 15140-122, LOT: 2009152), and. 2.5 × 10^6^ cells were seeded with 15 mL medium in a T75 flask and incubated at 37 °C with 5% CO_2_. 

For experiments, BeWo and SGHPL-4 cells were seeded in 6-well plates (400,000 cells/well) and 12-well plates (12,000 cells/well), respectively. The treatment started one day after seeding. The cells were treated with indicated concentrations of rosiglitazone (Sigma-Aldrich, cat. no. R22408), candesartan (Selleckchem, cat. no. S2037), or with vehicle control DMSO (Carl Roth, EG-no. 200-664-3, LOT: 450299516) for 48 h. After 24 h of incubation, the medium as well as the treatments were renewed.

For immunofluorescence, BeWo cells were seeded in chamber slides (150,000 cells/well). 

### 4.2. Explant Culture

First-trimester placentas were obtained through legal abortions before the 12th week of gestation after donors had signed informed consent. Inclusion criteria were a maternal age between 18 and 35 years, and a maternal BMI < 25. Explants with a size of approximately 3–5 mm were dissected from the villous chorion within 1 to 4 h after surgical termination of pregnancy. The explants were cultured in DMEM (1×) Dulbecco´s Modified Eagle Medium (Gibco, cat. no. 31885-023, LOT: 1906052) containing 10% fetal bovine serum (HyClone^TM^, cat. no. SV30160.03, LOT: RB35939), and 5% penicillin–streptomycin (Gibco, cat. no. 15140-122 LOT: 2145455) under 2.5% O_2_ and 5% CO_2_ using a hypoxic work station (BioSpherix, Redfield, NY, USA). 

Explants were treated with either 100 µM rosiglitazone, 10 µM candesartan, or DMSO for control for 24 h. 

### 4.3. Protein Analysis

Explants and cells were lysed with 50–150 µL RIPA buffer containing 1× PhosSTOP EASYpack (Roch, ref. 04906837001, LOT: 26920800) and 1× complete Tablets EASYpack (Roche, ref. 04693116001, LOT: 26424900). The samples were stored at −80 °C until further processing. Explants were homogenized with the TissueLyser LT (Qiagen) and Stainless Steel Beads (5 mm, Qiagen). After tissue lysis samples were sonified with a Bioruptor^®^ Pico sonication device (Diagenode) for 10 cycles lasting 10 s each at 4 °C. Explant samples were then centrifuged at 8000 rpm for 10 min at 4 °C. The protein content was quantified via a Lowry protein assay.

For western blot, 15 µg protein was used. Samples were prepared with an LDS sample buffer (4×) (Invitrogen NuPAGE^®^, cat. no. NP0007, LOT: 1887691) and sample reducing agent (10×) (Invitrogen NuPAGE^®^, cat. no. NP0004, LOT:1771572). For electrophoresis, NuPAGE^TM^ 10% Bis-Tris Gel 1.0 mm × 10 well (Invitrogen, cat. no. NP0301BOX, LOT: 18061310) gels were used. For the transfer to the membrane, we used 40 mL of transfer buffer (20×) (Novex^®^ NuPAGE^®^, cat. no. NP0006-1, LOT: 1934554,) with 160 of methanol (Carl Roth^®^, cat. no. 4627.5, LOT:258272520, EG-no. 2006596, CAS:67-56-1) and 600 mL ddH_2_O. After the transfer, the membrane was stained with Ponceau S solution (Sigma^®^ Life Science, LOT: SLBR3445V, CAS: 6226-79-5) and blocked with a 5% milk-TBST (Carl Roth ^®^, cat. no. T145.2, LOT: 367253531) for one hour. The membrane was incubated with the primary antibodies at 4 °C overnight. PPARγ (concentration 1:1000, Santa Cruz Biotechnologies, ref. no. sc-7273, LOT: J1618) and β-Actin (concentration 1:250.000, abcam, cat. no. ab6276, LOT: GR311395-5) antibodies were used as primary antibodies. The membranes were washed and incubated with secondary antibodies Pierce Goat Anti-Mouse IgG (H+L) peroxidase-conjugated (Thermo Scientific, cat. no. 31430, LOT: WA319701) or Pierce Goat Anti-Rabbit igG (H+L) peroxidase-conjugated (Thermo Scientific, cat. no. 31460, LOT: WJ326770) for two hours at RT. For detection, WesternBright Quantum (Biozym, ref. no. 541015, LOT: 220204-73) was used. Images were acquired with iBright CL 1000 Imaging System (Thermo Fischer Scientific) and band densities were analyzed with Image Studio Lite 5.2. Results are presented as a ratio of the target protein and β-Actin band densities.

### 4.4. RNA Analysis

The PeqGOLD Total RNA Kit (C-Line) (peqGOLD VWR, cat. no. 12-6634-02, LOT: 080818-3) was used for RNA isolation. Following the isolation, RNA was transcribed to cDNA with the Reverse Transkriptions Kit (applied biosystems, Thermo Fisher, cat. no. 4368813, LOT: 01134894). For qPCR, 1 ng/µL cDNA combined with SYBR Green (Biozym, cat. no. 331416XL, LOT: B099621906) were used. Primers were bought from Microsynth and sequences are shown in Table 1. 

### 4.5. Immunofluorescence Staining and Imaging

Chamber slides were fixed in ice-cold acetone for 7 min, while antigen retrieval for paraffin sections was performed with citrate buffer (pH 6). For staining, the Ultravision LP Large Volume Detection System HRP Polymer kit (Thermo Scientific, cat. no. TL-125-HL, LOT: LHL190716) was used. Primary antibodies, PPARγ (dilution 1:100, St. John’s Laboratory, cat. no. STJ95201, LOT: 5201601) and E-cadherin (dilution 1:200, Cell Signaling, cat. no. 14472, LOT: 15), were diluted in antibody diluent (Dako, cat. no. S3022, LOT: 11206569). Incubation with primary antibodies was 30 min at room temperature (RT). Secondary antibodies, goat anti-rabbit IgG (H+L) cross-adsorbed secondary antibody, Alexa Fluor™ 555 (dilution 1:200, Thermo Scientific, cat. no. A-21428), and goat anti-mouse IgG (H+L) cross-adsorbed secondary antibody, Alexa Fluor™ 488 (dilution 1:200, Thermo Scientific, cat. no. A-11001) were diluted in PBS and incubated for 30 min at RT. Nuclei were stained with DAPI.

Stained slides were scanned with an Olympus VS200 slide scanner and analyzed with the image analysis software Visiopharm, version 2021.09. For the image analysis, the whole sample on the slide was divided into quadrants, which were analyzed separately. The FITC channel was used to set a threshold for the detection of villi regions. This threshold allowed separating villi objects from intervillous space. When holes within the villi objects of up to 500 µm^2^ in size were detected, the holes were closed.

E-cadherin staining in FITC was used to detect the trophoblast layer by the use of a polynomial local linear filter. This filter enhanced linear structures in the FITC channel. A threshold was then applied to this enhanced image, which allowed us to separate the trophoblast layer objects from the rest of the tissue.

Nuclei were detected and separated using the commercial Visiopharm app ‘Nuclei Detection, AI (Fluorescence)’ after villi and trophoblast detection. Nuclei were then classified as positive or negative. Therefore, a threshold of 70 (on an 8-bit scale of 0–255) was set on the PPARγ marker within the nucleus area. Positively classified cells were counted on the entire villous area as well as the trophoblast area.

### 4.6. Chicken Chorioallantoic Membrane (CAM) Assay

The *ex ovo* chicken chorioallantoic membrane assay was performed by using a collagen–nylon grid model according to Deryugina and Quigley [63]. Fertilized white Lohmann chicken eggs (Schropper, Gloggnitz, Austria) were washed and incubated at 37.6 °C and 60% humidity (Incubator Easy200, J. Hemel Brutgeräte, Germany). After 3 days of incubation, the eggs were cracked, and the embryos were placed on sterile weighing boats and covered with square Petri dishes. On day 10 of the embryonic development, collagen–nylon grid on-plants containing 0.1 µmol/L angiotensin II (Sigma-Aldrich, Merck, Vienna, Austria) and DMSO, respectively, were placed on the CAM for 72 h (6 eggs with 6 on-plants each). Newly formed blood vessels were counted manually with a stereomicroscope (Olympus, Japan). 

### 4.7. LDH Assay

To determine the LDH concentration in the supernatants of cells, the LDH Cytotoxicity Detection Kit (Takara, cat. no. MK401) was used according to the manufacturer’s instructions.

### 4.8. Leptin ELISA

We used the Human Leptin Quantikine ELISA Kit (R&D Biosystems, cat. no. DLP00) according to the manufacturer’s instructions to determine the leptin concentration in supernatants.

## 5. Conclusions

Candesartan is known to be teratogenic in the second half of pregnancy, but evidence for its effects in the first trimester is scarce.

Trophoblast cells that lack the classical candesartan target–receptor AGTR1, express the PPARγ receptor, a potential target for AGTR1 antagonists. PPARγ is essential for the proper development of the placenta and, thus, for the entire course of pregnancy, despite the expression of this receptor in trophoblast. However, we could not find any association between candesartan exposure and PPARγ activation in placental trophoblasts in this study.

Even though treatment with candesartan led to the increase of the *PPARG* target leptin in first-trimester placental villous explants, all other results point to a lack of candesartan interactions with the trophoblast layer itself, while rosiglitazone treatment of trophoblast cells influenced the expressions of several PPARγ target genes.

In conclusion, this study adds biological plausibility to the scarce observational data indicating an absence of substantial adverse effects of early pregnancy exposure to AGTR1 antagonists on placental development. This is especially important since randomized controlled trials that include pregnant women are not feasible due to substantial ethical concerns.

From this perspective, acknowledging the various limitations of in vitro experiments in predicting teratogenicity in humans, hypothesizing, exploring, and occasionally refuting potential teratogenic mechanisms can contribute to teratologic risk assessment.

However, while a lack of candesartan–interference with trophoblast via PPARγ receptors is quite reassuring, such an interaction was observed for the oral antidiabetic drug rosiglitazone. In addition, it remains an open question whether candesartan can interact relevantly with placental *AGTR1*-expressing myofibroblasts and potentially influence placental or even fetal vascularization.

## Figures and Tables

**Table 1:** Primer sequences.

Primer	Sequence
TBP	F: 5‘-TGA CCC AGC ATC ACT GTT TC-3’ R: 5’-CCA GCA CAC TCT TCT CAG CA-3’
ACTB	F: 5’-AAA GAC CTG TAC GCC AAC AC-3’ R: 5’-GTC ATA CTC CTG CCT GCT GAT-3’
B2M	F: 5’-GAT GAG TAT GCC TGC CGT GT-3’ R: 5‘-TGT CTC GAT CCC ACT TAA CTA TCT-3’
HPRT1	F: 5’-GAA AGG GTG TTT ATT CCT CAT GAA-3’ R: 5’-CAA GCA GGT CAG CAA AGA ATT T-3’
YWHAZ	F: 5’-GGT GGC CAA TAT GGG GAT GT-3’ R: 5‘-TCC CTT TTA TTC CCC GCC AG-3’
GAPDH	F: 5’-ACC CAC TCC TCC ACC TTT-3’ R: 5’-CTG TTG CTG TAG CCA AAT-3’
LEP	F: 5’-GTG CGG ATT CTT GTG GCT TT-3’ R: 5’-AGG AGA CTG ACT GCG TGT GT-3’
FABP4	F: 5’-AAG GCG TCA CTT CCA CGA GAG-3’ R: 5‘-AAT GCG AAC TTC AGT CCA GGT-3‘
CEBPα	F: 5’-CTT GTG CCT TGG AA-3’ R: 5’-GCT GTA GCC TCG GAA AGG A-3’
CD34	F: 5’-CGG CCA TTC AGC AAG ACA AC-3’ R: 5’-TAG CAC GTG GTC AGA TGC AG-3‘
PPARG1	F: 5’-AAG GCC ATT TTC TCA AAC GA-3’ R: 5‘-AGG AGT GGG AGT GGT CTT CC-3‘
PPARG2	F: 5’-CCA TGC TGT TAT GGG TGA AA-3’ R: 5‘-TCA AAG GAG TGG GAG TGG TC-3‘
hMMP1	F: 5‘-CTC TGG AGT AAT GTC ACA CCT CT-3’ R: 5‘-TGT TGG TCC ACC TTT CAT CTT C-3’
AGTR1	F: 5‘-GCG CGG GTT TGA TAT TTG ACA-3’ R: 5‘-TCA AAT ACA CCT GGT GCC GA-3’

## Data Availability

The data presented in this study are available on reasonable request from the corresponding author.
